# Survey of Tuberculosis Hospitals in China: Current Status and Challenges

**DOI:** 10.1371/journal.pone.0111945

**Published:** 2014-11-03

**Authors:** Jian Du, Yu Pang, Yuhong Liu, Fengling Mi, Shaofa Xu, Liang Li

**Affiliations:** 1 Beijing Chest Hospital, Capital Medical University, Beijing, China; 2 Beijing Tuberculosis and Thoracic Tumor Research Institute, Beijing, China; 3 Administration Office, Clinical Center on Tuberculosis, China CDC, Beijing, China; 4 National Center for Tuberculosis Control and Prevention, Chinese Center for Disease Control and Prevention, Beijing, China; McGill University, Canada

## Abstract

**Background:**

Hospitals will play an increasingly important role in delivering TB services in China, however little is known in terms of the current landscape of the hospital system that delivers TB care.

**Methodology/Principal Findings:**

In order to examine the status of TB hospitals we performed a study in which a total of 203 TB hospitals, with 30 beds or more, were enrolled from 31 provinces and Xinjiang Production and Construction Corps. Of the 203 hospitals, 93 (45.8%) were located in the eastern region of China, 84 (41.4%) in the central region, and 26 (12.8%) in the western region, while there were 34.6 million TB patients in western China, accounting for 34.6% of the TB burden nationwide. The total number of staff in these 203 hospitals was 83,011, of which 18,899 (22.8%) provided health services for TB patients, (physicians, nurses, lab technicians, etc). Although both the overall number of the health care workers and TB staff in the 203 hospitals increased from the year 1999 to 2009, the former increased by 52%, while the latter increased only by 34%, showing that the percentage of TB staff declined significantly (χ^2^ = 181.7, *P*<0.01). The total annual income of the 203 hospitals increased 5.5 fold from 1999 to 2009, while that from TB care increased 3.8 fold during the same period. TB care and control experienced a relatively slower development during this period as shown by the lower percentage of TB staff and the lesser increase in income from TB care in the hospitals.

**Conclusions/Significance:**

In conclusion, our findings demonstrated that hospital resources are scarcer in western China as compared with eastern China. In view of the current findings, policymakers are urged to address the uneven distribution of medical resources between the underdeveloped west and the more affluent eastern provinces.

## Introduction

Tuberculosis (TB) is still a global health problem [1],[2]. According to the estimation by the World Health Organization (WHO), there were 8.6 million new TB cases and 1.3 million people died from TB in 2012 [2]. China has the world's second largest tuberculosis burden, only behind India, with more than 1 million new cases of tuberculosis every year [3], [4]. To achieve the global tuberculosis control target, several effective strategies have been implemented in China since the 1990s, including the WHO-recommended DOTS strategy and strengthening a health insurance system for TB [5], [6]. Through these efforts, the prevalence of smear-positive tuberculosis decreased from 170 cases to 59 cases per 100 000 population from 1990 to 2010 [7].

In China, there are two systems providing clinical services for tuberculosis patients, the public health system and the hospital system. On the one hand, the public health system is entirely funded by the government, which is responsible for providing a free first-line anti-TB regimen for TB patients and the management of TB patients during the follow-up period. In contrast, the hospital system is only partially funded by the government and in-patients and out-patients are charged for diagnosis and treatment with partial coverage by their health insurance. Despite significant achievements, a major challenge for the national tuberculosis programmes (NTPs) in China is that approximately 60% of TB patients seek initial health care in hospitals rather than at public health centers [7]. In order to overcome this challenge, the Ministry of Health of China has recently formulated a new strategy for TB, to strengthen cooperation between the hospital system and the public health system [8]. Based on the design of this new model, the diagnosis and treatment for TB patients will be completed in TB specialized hospitals chest/pulmonary hospitals, infectious disease hospitals, and general hospitals with a TB department and general hospitals with TB departments, while the follow-up function is reserved for the public health system. Hospitals will therefore play an important role in delivering TB services in China in the future [9]. There is currently limited information and knowledge regarding the hospital system as it pertains to TB care.

In the present study, we conducted the first survey examining TB hospitals in China. Our aim was to investigate the current status of TB hospitals, including distribution, human resources, and other characteristics, and to summarize the ongoing challenges found in the TB hospital system.

## Methods

### Ethics Statement

The protocols applied in this study were approved by the Ethics Committee of the Beijing Chest Hospital, affiliated to Capital Medical University. The participants provided their written informed consent to participate in this study.

### Setting and Data Collection

The study was conducted in 31 provinces and Xinjiang Production and Construction Corps of China, in 2010. All hospitals with 30 beds or more for TB were enrolled, including TB specialized hospitals, pulmonary hospitals, chest hospitals, hospitals for infectious diseases, and general hospitals with TB departments. The questionnaires were prepared by staff of the National TB Clinical Center, including information on the founding year, geographic distribution, classification, human resources, income and expenditures, etc. The provincial health administration was in charge of distributing questionnaires to the local TB hospitals. After the questionnaires were filled out by the TB hospital staff, they were delivered to the National TB Clinical Center.

All staff, who were responsible for filling out the questionnaires, attended the training course held by the Clinical Center on Tuberculosis, China CDC. In addition, 20% of the questionnaires were checked against the original hospital records by staff from the provincial bureau of health. Any discrepant data were resolved by telephone interview.

### Definitions

All hospitals in China are classified into one of three levels: tertiary, secondary or primary. Tertiary hospitals are hospitals with more than 500 beds, secondary hospitals have 100–499 beds, and primary hospitals have less than 100 beds [10].

TB specialized hospitals were defined as hospitals that provide clinical service for only tuberculosis, rather than other infectious diseases. The infectious disease hospital was defined as hospitals that could provide clinical service for tuberculosis and other infectious diseases, including HIV, hepatitis, etc. The general hospital with a TB department was defined as general hospitals that included a department with no less than 30 beds designated for TB.

According to the Chinese administrative division, the Eastern region of China includes 12 provinces: Liaoning, Hebei, Beijing, Tianjin, Shandong, Jiangsu, Zhejiang, Shanghai, Fujian, Guangdong, Guangxi and Hainan; the Central region of China includes 9 provinces: Heilongjiang, Jilin, Inner Mongolia, Shanxi, Henan, Hubei, Jiangxi, Anhui and Hunan; the Western region of China includes 10 provinces: Shaanxi, Gansu, Qinghai, Ningxia, Xinjiang, Sichuan, Chongqing, Yunnan, Guizhou and Xizang.

### Analysis

All the data were double-entered by two persons into Epi-Info software (Atlanta, GA). The chi-square test was performed to compare the distribution among different characteristics in SPSS 15.0. The difference was considered as significant when *P* value was less than 0.05.

## Results

### Distribution of TB Hospitals

**Figure 1:**
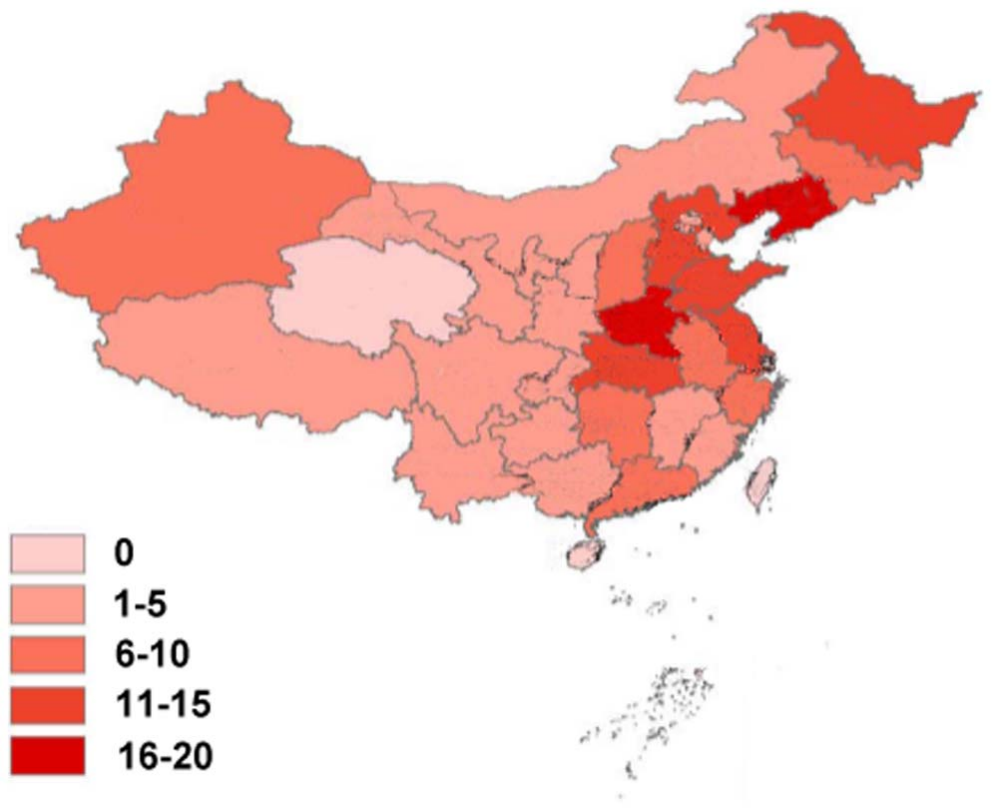
Distribution of TB hospitals among different provinces. The numbers represent the number of hospital in different provinces.

A total of 203 TB hospitals were enrolled from 31 provinces and Xinjiang Production and Construction Corps in this study, including 26 (12.8%) provincial hospitals and 177 (87.2%) prefectural hospitals. Out of 31 provinces, Henan and Liaoning had more than 16 TB hospitals, while Qinghai and Hainan had no TB hospitals with 30 beds or more for TB. In addition, 93 (45.8%) hospitals were located in the eastern region of China, 84 (41.4%) in the central region, and 26 (12.8%) in the western region. The 203 hospitals together comprised a total of 71,457 beds, of which 23,962 (33.5%) beds were designated for TB. Among the 23,963 TB beds, 10,804 (45.1%) were located in eastern China, 9611 (40.1%) in central China, and 3547 (14.8%) in western China (Table 1 & Fig. 1).

**Table 1:** Distribution of TB hospitals and beds designated for TB among different provinces.

Region	Province	No. of TB hospitals	No. of beds designated for TB
Eastern	Liaoning	16	3607
	Hebei	15	1199
	Shandong	14	1540
	Jiangsu	12	1069
	Guangdong	9	972
	Zhejiang	8	516
	Shanghai	7	479
	Guangxi	5	524
	Beijing	3	416
	Fujian	2	285
	Tianjin	2	197
	Hainan	0	0
Central	Henan	20	1586
	Heilongjiang	13	2219
	Hubei	12	967
	Jilin	10	1342
	Shanxi	9	975
	Hunan	6	909
	Anhui	6	706
	Jiangxi	5	370
	Inner Mongolia	3	537
Western	Xinjiang	7	934
	Guizhou	5	435
	Shaanxi	3	920
	Chongqing	3	423
	Yunnan	3	390
	Xizang	2	62
	Gansu	1	208
	Sichuan	1	100
	Ningxia	1	75
	Qinghai	0	0
	Xinjiang Production and Construction Corps	0	0
Total		203	23962

In addition, we compared the TB prevalence and TB hospital resources amongst the different regions of China. The number of pulmonary TB patients was estimated according the data from the fifth national tuberculosis prevalence survey [10] and the sixth nationwide population census conducted in 2010 (http://www.stats.gov.cn/ztjc/zdtjgz/zgrkpc/dlcrkpc). As shown in Table 2, there were 34.6 million TB patients in western China, accounting for 34.6% of the TB burden nationwide, while only 12.8% of hospitals and 14.8% of beds were located in that region (Table 2).

**Table 2:** Comparison of TB epidemics and TB hospital resource among different regions of China.

Region	No. of TB hospital (%)	No. of beds designated for TB (%)	No. of pulmonary TB patients (million)^a^ (%)
Eastern	93(45.8)	10804(45.1)	1.734(29.8)
Central	84(41.4)	9611(40.1)	2.071(35.6)
Western	26(12.8)	3547(14.8)	2.013(34.6)

a The number of pulmonary TB patients was estimated according to the data from the fifth national tuberculosis prevalence survey and the sixth nationwide population census conducted in 2010.

### Classification of TB Hospitals

**Figure 2:**
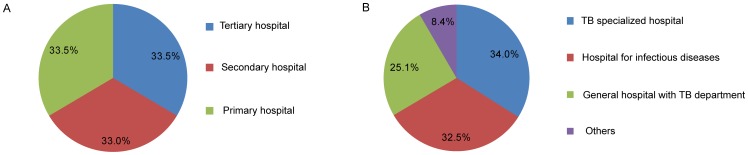
Classification of TB hospitals.

Sixty-eight (33.5%) of 203 hospitals were tertiary hospitals, and 67 (33.0%) and 68 (33.5%) were secondary and primary hospitals respectively. When these hospitals were further distinguished by hospital designations, there were 69 (34.0%) TB specialized hospitals. In addition, 66 (32.5%), 51 (25.1%) and 17 (8.4%) hospitals were classified as hospitals for infectious diseases, general hospitals with TB departments, and others respectively (Fig. 2).

### Human Resources

**Figure 3:**
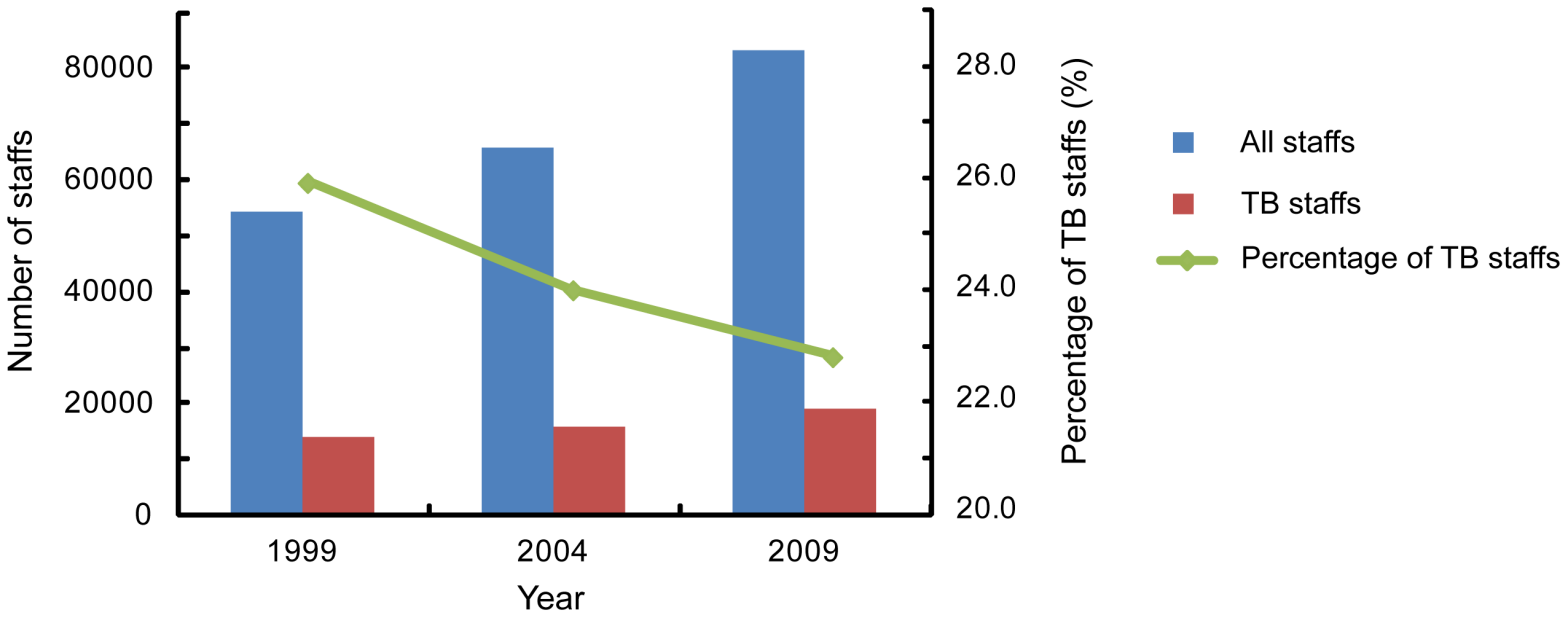
The numbers of staff and TB stafffrom 1999 to 2009.

**Figure 4:**
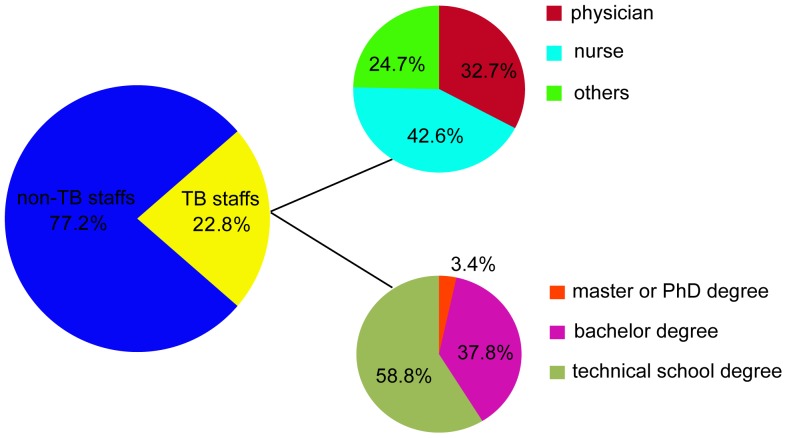
Composition of TB staff.

The total number of staff in these 203 hospitals was 83,011, of which 18,899 (22.8%) provided health services for TB patients. Among all the TB staff, there were 6,181 (32.7%) physicians, 8,053 (42.6%) nurses and 4,655 (24.7%) other staff (such as lab technicians) (Fig. 3&4).

When examining educational backgrounds, 3.4 percent of TB staff had a master or PhD degree, while 58.8% had an educational background lower than a bachelor degree (Fig. 4). Of the 6,181 physicians, there were 465 (7.5%) physicians with a master or PhD degree, 4079 (66.0%) with a bachelor degree and 1637 (26.5%) with an educational background lower than a bachelor degree, who were less qualified physicians, In contrast, the proportion of master/PhD degrees and bachelor degrees was 0.3% (27/8053) and 15.9% (1278/8053) among the nursing group, Statistical analysis revealed that the distribution of staff with different education backgrounds showed a significant difference between the physician and nurse groups (χ^2^ = 4806.8, *P*<0.01). We also analyzed the dynamic change in human resources between 1999 and 2009. As shown in Fig. 3, although both the absolute numbers of all health care workers and TB staff in the 203 hospitals increased during this period, – the overall number increased by 52%, and the TB staff component increased only by 34%, so that the percentage of TB staff declined by 12.3%, from 25.9% in 1999 to 22.8% in 2009, a significant decrease (χ^2^ = 181.7, *P*<0.01).

### Income and Expenditure

**Figure 5:**
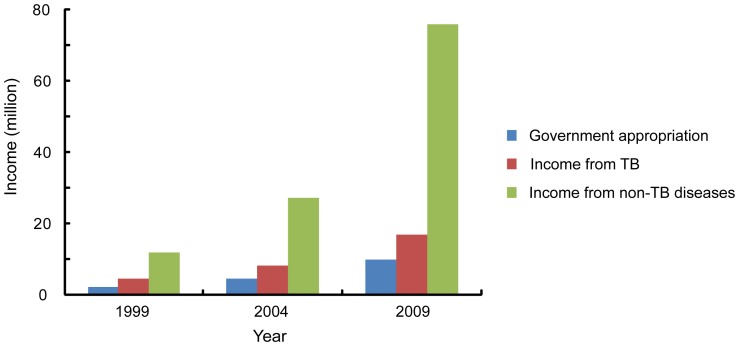
Income of TB hospitals from 1999 to 2009.

In 2009, the average of the government appropriation for TB hospitals was 10.01 million, accounting for 9.7% of the average annual income in a hospital. The income from TB diagnosis and treatment was 16.95 million (16.5%), while that from other diseases was 75.85 million (73.8%). The total annual income of the 203 hospitals increased 5.5 fold from 1999 to 2009 while the income from TB care only i increased 3.8 fold (Fig. 5). If the overall inflation rate in China from 1999 to 2009 was considered (http://data.worldbank.org), the total annual income and the income from TB care increased 3.9 and 2.7 fold, respectively.

## Discussion

In the recent 10 years, China has successfully curbed the spread of tuberculosis [7]. The marked improvement in tuberculosis control was mainly driven by the enhanced capacity of the public health system [5], [7]. Along with the reformation of TB diagnosis and treatment, hospitals will play a more critical role in the control of TB in China [9]. In comparison with the public health system, TB hospitals have numerous inherent advantages such as provision of comprehensive clinical treatment and enhanced laboratory settings. Nevertheless, our research showed that several problems lie in the TB hospital system, which may have potential challenges for the prevention and control of TB. First, hospital resources are significantly scarcer in western compared with eastern China. However, the National Tuberculosis Prevalence Survey in China has demonstrated that western China has the highest prevalence of tuberculosis [7]. Notably, the gap between western and non-western areas in tuberculosis prevalence has widened from 1990 to 2000. The unbalanced distribution of hospital resources between these two areas makes it difficult to access health services for the rural regions which have the highest incidence of TB in western China. In view of the slower economic development in western China, policymakers need to promote tuberculosis control efforts in this area, including improved financial resources and technical training. Furthermore, it is essential to establish a collaboration mechanism between western and eastern areas to improve the current unequal situation.

Despite the fact that income from TB care in the hospitals increased from 1999 to 2009, the proportion of income from TB care compared with the overall income, decreased by more than 7.54%(from 24.03% to 16.49%), indicating the relatively slower development in TB services in the last 10 years. Due to the small proportion of hospital income from government appropriation, hospitals have to face the burden of covering their costs. When compared with TB, other diseases, such as cardiology and thoracic surgery, bring a higher economic return, indicating that TB care may be neglected. Therefore, a reasonable and increased economic investment in TB control is essential to motivate health providers to provide medical services for TB. The insufficient financial support for TB hospitals was also reflected in increased personal medical expenses for TB patients from 1999 to 2009. Although more than half of the inpatient fee can be covered by health insurance, TB patients have to suffer from a high economic burden, which may result in the reluctance of patients to seek medical care, and by extension, delayed anti-TB treatment and continued TB transmission in the community.

Human resources play a unique and central role in TB medical care [10]. Our data reveal that the challenges related to TB health care in China include a shortage of qualified staff. TB physicians prefer to transfer to other departments rather than the TB department, because of low salaries. In addition, unsatisfactory working condition and inadequate TB infection control measures result in health care workers being at a high risk of TB infection [11] adding to the crisis in human resources. According to the Guideline of the Ministry of Health, the nurse–physician ratio should be no less than 2∶1 [12]. However, this ratio in TB hospitals was only 1.3∶1 in this study, indicating that human resources problems in nursing exist in the current setting. In addition, our study revealed that most nurses graduated from technical school rather than college. Several publications show that higher nurse-to-patient ratios and the higher proportion of nurses with a baccalaureate level education are associated with lower mortality rates [13], [14], [15]. Our findings highlight the urgent need to improve the quantity and education level of nurses in order to provide more accessible and satisfactory health services.

There are several limitations in this study. First, this survey did not collect data from hospitals with less than 30 beds, which will result in an underestimation of the resources of TB hospitals in China. Secondly, the length of employment of health care workers, an important indicator of stability in human resources, was not included in this survey. Nevertheless, this survey provides an important insight into TB hospitals in China in order to promote improvements in the effective TB control strategy in China.

In conclusion, our findings demonstrate that hospital resources, including financial resources and well-trained health care workers, are significantly lower in western as compared to eastern China. Despite the increased income from TB care between 1999 and2009, the proportion of TB income compared to the overall hospital income decreased, indicating that TB services had a relatively slower development over the last 20 years. In addition, the low nurse-to-physician ratio and low staff education levels highlight the urgent need to improve the quantity and education level of nurses to provide more accessible and satisfactory health services. In view of the current setting, policymakers should focus on improving the medical resources of TB hospitals in western China.
